# New Resources on Transplantation Safety

**Published:** 2013-06-28

The U.S. Department of Health and Human Services has released the 2013 *PHS Guideline for Reducing Human Immunodeficiency Virus, Hepatitis B Virus, and Hepatitis C Virus Transmission Through Organ Transplantation* (1), available online at http://www.publichealthreports.org/issueopen.cfm?articleID=2975. This guideline updates the 1994 U.S. Public Health Service (PHS) *Guidelines for Preventing Transmission of Human Immunodeficiency Virus Through Transplantation of Human Tissue and Organs* (2).

Major changes from the 1994 PHS guidelines are that the 2013 PHS guideline includes 1) recommendations that organ donors be screened for hepatitis B virus (HBV) and hepatitis C virus (HCV) in addition to human immunodeficiency virus (HIV); 2) recommendations for new, more sensitive laboratory testing; and 3) inclusion of a revised set of risk factors for HIV, HBV, and HCV infection. CDC’s *Solid Organ Transplantation and the Probability of Transmitting HIV, HBV, or HCV: A Systematic Review to Support an Evidence-Based Guideline* (3) comprises the primary evidence underlying the recommendations in the 2013 PHS guideline.

In addition, CDC recently launched a website devoted to transplant safety, which features 1) the role of CDC and other agencies and organizations involved in organ and tissue safety; 2) donor screening and testing requirements; and 3) information for health-care providers. The website can be accessed at http://www.cdc.gov/transplantsafety.
